# miRNAs, Mesenchymal Stromal Cells and Major Neoplastic and Inflammatory Skin Diseases: A Page Being Written: A Systematic Review

**DOI:** 10.3390/ijms24108502

**Published:** 2023-05-09

**Authors:** Mariangela Di Vincenzo, Federico Diotallevi, Silvia Piccirillo, Gianluca Carnevale, Annamaria Offidani, Anna Campanati, Monia Orciani

**Affiliations:** 1 Department of Clinical and Molecular Sciences—Histology, Università Politecnica delle Marche, 60126 Ancona, Italym.orciani@univpm.it (M.O.); 2 Department of Clinical and Molecular Sciences—Dermatological Clinic, Università Politecnica delle Marche, 60126 Ancona, Italy; 3 Department of Biomedical Sciences and Public Health—Pharmacology, Università Politecnica delle Marche, 60126 Ancona, Italy; 4 Department of Surgery, Medicine, Dentistry and Morphological Sciences with Interest in Transplant, Oncology and Regenerative Medicine, Università di Modena e Reggio Emilia, 41121 Modena, Italy

**Keywords:** miRNAs, human mesenchymal stromal cells, skin disorders

## Abstract

Micro RNAs (miRNAs) are a type of non-coding RNA (ncRNA) and typically interact with specific target mRNAs through complementary base pairing, affecting their translation and/or stability. MiRNAs regulate nearly all cellular functions, including the cell fate of mesenchymal stromal cells (MSCs). It is now accepted that various pathologies arise at the stem level, and, in this scenario, the role played by miRNAs in the fate of MSCs becomes of primary concern. Here we have considered the existing literature in the field of miRNAs, MSCs and skin diseases, classified as inflammatory (such as psoriasis and atopic dermatitis-AD) and neoplastic (melanoma and non-melanoma-skin-cancer including squamous cell and basal cell carcinoma) diseases. In this scoping review article, the evidence recovered indicates that this topic has attracted attention, but it is still a matter of opinion. A protocol for this review was registered in PROSPERO with the registration number “CRD42023420245”. According to the different skin disorders and to the specific cellular mechanisms considered (cancer stem cells, extracellular vesicles, inflammation), miRNAs may play a pro- or anti-inflammatory, as well as a tumor suppressive, or supporting, role, indicating a complex regulation of their function. It is evident that the mode of action of miRNAs is more than a switch on–off, and all the observed effects of their dysregulated expression must be checked in a detailed analysis of the targeted proteins. The involvement of miRNAs has been studied mainly for squamous cell carcinoma and melanoma, and much less in psoriasis and AD; different mechanisms have been considered, such as miRNAs included in extracellular vesicles derived both from MSCs or tumor cells, miRNAs involved in cancer stem cells formation, up to miRNAs as candidates to be new therapeutic tools.

## 1. Introduction

microRNAs (miRNAs) are small non-coding RNAs that span between 18 and 24 nucleotides [1,2], playing a central role in gene expression on a post-transcriptional level: miRNAs interact with complementary sequences of the 3′untranslated region (UTR) of mRNAs through base-pairing, inducing mRNA degradation and translational repression.

miRNAs are well conserved in eukaryotic systems and are believed to be an essential and evolutionary ancient component of gene regulatory networks: they influence many cellular functions, such as development, differentiation, growth and metabolism [3,4].

More recently, it has been reported that miRNAs regulate stemness, self-renewal and differentiation in embryonic and adult stem cells, such as mesenchymal stromal cells (MSCs) [5,6,7,8], determining their fate.

MSCs are multipotent cells residing in multiple tissues with the capacity for self-renewal and differentiation into various cell types [9,10]. Our previous works confirmed that different pathologies, ranging from the dermatologic to the fibrotic ones [11,12,13,14,15,16,17,18,19,20], arise at the staminal level: MSCs show the same dysregulated pathological properties found in differentiated cells, opening up the hypothesis that the onset of the diseases could be backdated at the stem level.

How miRNA dysregulation could affect the behavior of MSCs and, in turn, lead to the onset of selected diseases is a matter of growing interest and some manuscripts dealing with this topic are emerging [3,21,22,23,24,25,26,27].

The most frequent skin disorders may be sub-grouped into inflammatory (such as psoriasis and atopic dermatitis—AD) and neoplastic (melanoma and non-melanoma-skin-cancer, including squamous cell and basal cell carcinoma) diseases.

This scoping review article, aimed to provide a broad overview of what is known about the effects of dysregulated miRNAs on MSCs and, in turn, the onset of the selected skin disorders (Supplementary Figure S1), was based on the approach developed by Arksey and O’Malley [28].

## 2. Results

**Figure 1:**
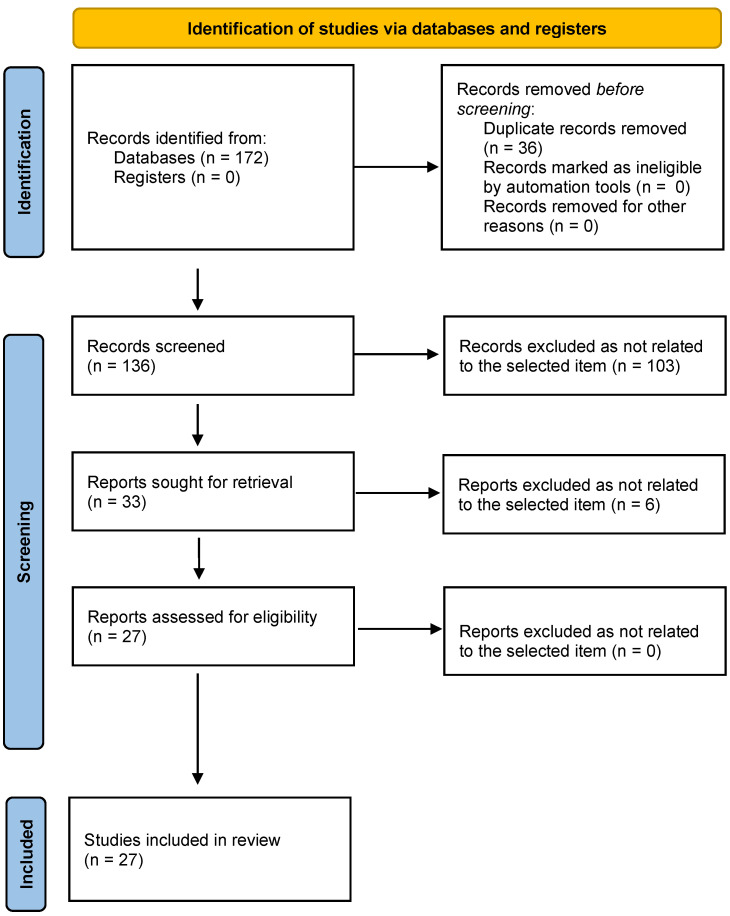
Preferred reporting items for reviews on miRNA expression in MSCs involved in skin diseases.

The flowchart of the PRISMA study is shown in Figure 1.

Starting from the initial selectin of 171 records, our search identified 135 records after removing duplicates. After the review of the titles and abstracts, 102 citations were dropped (reports not strictly related to the item retrieved) and 33 were evaluated for full-text eligibility. After review of the full text, 27 pre-clinical trials, controlled clinical trials, case-control studies, cross-sectional studies and case series were found to be eligible and included in this study.

The data found demonstrated that the role of miRNA expression in MSCs driving skin diseases is a key requirement for understanding the future positioning of regenerative medicine in this field.

### 2.1. Neoplastic Diseases

#### 2.1.1. Melanoma

The nine selected articles, while answering the research question on the relationship between miRNAs and MSCs, and on how this interaction could influence the onset and progression of melanoma, use very diversified approaches and consider different cellular mechanisms.

Wang et al. [29] and Yang et al. [30] focused on the role played by MSCs-derived EVs, containing miRNAs that affect the progression of melanoma. The considered miRNAs are different, miR-138-5p and miR-374a-5p in the manuscripts respectively by Wang et al. [29] and Yang et al. [30]. Authors found that elevated miR-138-5p levels in EVs reflected increased cell apoptosis, restraining melanoma progression; this effect was directly driven by *SOX4*, a stemness marker targeted by miR-138-5p.

According to Yang et al. [30], the inhibition of miR-374a-5p by *NEAT1* was requested to promote the expression of macrophage M2 markers, which, in turn, enhanced the malignancy of melanoma cells. These two manuscripts suggest that miRNAs inside MSCs-derived EVs may reduce the malignancy of melanoma.

Gyukity-Sebestyén considered the effects of melanoma-derived EVs on MSCs [31]. The authors found that, after internalization, EVs induced a malignant-like transformation of MSCs by cell-molecular oncogenic reprogramming of target cells. The observed “MSC re-education” could be attributable to EV-transported miRNAs and molecules that controlled the key mechanisms of cancer progression.

Melanospheres are described as melanoma cancer stem cells (CSCs) firstly responsible for metastasis. Sahranavardfard et al. [32] identified miR-205, -15b, -203 and -9 as linked to melanoma status and its pluripotency: these miRNAs were dysregulated in all groups of cell line melanospheres and affected the expression of genes related to stemness (such as *OCT4*, *NANOG*, and *SOX2*), with the effect of promoting the proliferation and motility of malignant cells.

An increase in stemness potential and furthering of the metastatic process has also been reported by Fomeshi et al. [33], evaluating melanospheres overexpressing miR-10b, 21, 200c, 373 and 520c.

The same effects were addressed to miR-203 in another manuscript by Sahranavardfard et al. [34]; the authors defined this miRNA as oncomiR in melanoma cells with a higher ability for repopulation, tumorigenicity, self-renewal and metastasis, and for preparing cancer cells for secondary seeding.

According to Wang et al. [35], suppression of miR-381-3p led to higher efficiency in melanosphere formation and it aggravated stemness, and, therefore, cell proliferation, migration and EMT in melanoma.

Divisato et al. [36] recalled the evidence for the tumor-suppressing properties of miR-29 family members; in particular, miR-29a, involved in the early differentiation of stem cells, could mediate antiproliferative effects on melanoma cells by downregulating *CDK6*, a regulator of the G1/S phase. miR-29 has been already classified as a tumor-suppressor miRNA for different tumors, but now the possibility to use synthetic miR-29 as a therapeutic tool seems closer for the treatment of melanoma.

Lastly, Maadi et al. [37] reported that transfection of melanoma cell lines with miR-302 caused a reprogramming process of these somatic cells and transformed them to a stem cell-like state. The miR-302 cluster modulated the malignant phenotype, leading to less invasive behavior and increased apoptosis of melanoma cells through a reprogramming process in which stemness plays a pivotal role and opening to innovative therapeutic tools.

The revision of items dealing with miRNAs, MSCs and melanoma refers to a condition of great complexity, characterized by the lack of well-defined miRNAs correlated to melanoma (only miRNA-203 has been considered by two manuscripts) and of a unique hypothesis about how miRNAs’ dysregulation can affect the behavior of MSCs, driving the onset of skin neoplastic disorders. While, on the one hand, miRNAs associated with MSC-derived EVs seem to play a role as suppressors, on the other hand, miRNAs of melanoma-derived EVs appear to have a less noble role, driving MSCs re-education toward a malignant-like phenotype. Oncomir properties have also been proposed for different miRNAs related to melanosphere formation, which positively affect proliferation and metastasis. At the same time, miR-29a and miR-302 are promising candidates for new therapeutic approaches.

This relationship has become evident as this research field has gained attention, but further studies are necessary to better identify both the mechanisms underlying this possible interaction and which miRNAs deserve more attention.

#### 2.1.2. Non-Melanoma Neoplastic Skin Disorders

##### Squamous Cell Carcinoma (SCC)

In 2021, Wang et al. reviewed the existing literature on the topic of EVs and head and neck (HN) SCC [38]. They reported that in vitro and in vivo experiments had previously indicated that the overexpression of miRNA-34c inserted in bone marrow-MSCs (BM-MSCs)-derived EVs inhibited malignant features such as invasion, migration, proliferation and EMT by targeting β-Catenin.

Similarly, MSCs-EVs-miRNA-185 significantly reduced cell proliferation and angiogenesis in oral (O) SCC tissue and induced apoptosis, indicating their potential role as a novel therapeutic option for OSCC. In addition, it was reported that co-cultures of cells from OSCCs with BM-MSCs-derived EVs containing a high level of miRNA-101-3p could prevent cell invasion and migration, blocking the progression of tumors.

The antitumoral role of miRNA-101-3p has also been reported in HNSCC where, according to Panvongsa et al. [39], BM-MSCs-derived EVs overexpressing miR-101-3p were able to suppress cancer cell proliferation and tumor growth both in vitro and in vivo by targeting the Collagen Type X Alpha 1 Chain gene (*COL10A1*), resulting in downregulation of Collagen X expression. In the same review, the ability of engineered MSC-EVs with a high copy number of miR-185 to reduce the severity of inflammation as well as the grade and number of dysplastic lesions was also outlined.

Eight manuscripts considered the effects of selected miRNAs on CSCs in different types of SCC.

Referring to HNSCC, both oncomiRNAs and tumor suppressor miRNAs have been reported [40].

Oncomirs involved in the stemness of HNSCCs have been divided according to their action mode in “gene expression” (miRNA-125, 134 negatively modulate the expression of p53 and E-cadherin, respectively), “suppressor inhibition” (miR-196b promotes cell proliferation, migration and invasion abilities by inhibiting *PCDH-17*) and “signal transduction” (miR-19a and miR-424 promote EMT by suppressing *TGFBR3*, miR-106A-5p inhibits autophagy and activates MAPK signaling by targeting *BTG3*, miR-629-3p enhances EMT via targeting *ESRP2*).

Among the miRNAs acting as tumor suppressors that are involved in the stemness of HNSCCs, the miRNA let-7 family and miRNA-520b have been held in high regard by Fitriana et al. [40]; the authors defined the miRNA let-7 family as controllers of normal cellular development and differentiation, and a reduction in let-7 led to an improvement of carcinogenesis through mechanisms related to stemness properties.

By suppressing let-7i expression, cells acquired stemness features by activating stem specific genes such as *POU5F1*, *NANOG* and *SOX2* via histone modifications.

Similarly, miR-520b silencing led to an acceleration of tumor growth, while its over-expression/administration hugely restrained tumorigenesis. miR-520b was able to suppress spheroid cell formation through the reduction of stemness regulators such as *NANOG* and *OCT4* and to sensitize cells to therapeutic drugs and irradiation.

Conversely, the upregulation of miR-21 has been associated with progression and resistance to therapy; in detail, its enhanced expression seemed to be the result of the direct activation of the stem cell markers *NANOG-STAT3*, which formed a complex able to bind to the promoter region of miR-21, enhancing its transcription [41].

Subramanian et al. [42] put the attention on several miRNAs (miRNA 15b, 16, 17, 107, 20a, 106b, 128, 425, 223, 299, 145, 302a, 494, 409 and 128) that, in HNSCC, target CSCs and confer them therapy resistance. The authors reported that novel C-terminal Hsp90 inhibitors could restore the aberrant expression of the selected miRNAs, sensitize cells to drugs and negatively affect tumor progression.

In the cutaneous (C) SCC, the existence of CSCs characterized by increased proliferation, inhibited apoptosis and the ability to sustain CSCC progression has been observed. This cell population showed high levels of miR-142-5p, which, in turn, was able to drive the overexpression of *CD44*, *SOX2*, *NANOG* and *OCT4*, underlining the importance of inducing stemness in CSCs [43].

Regarding OSCC, aberrant miRNA expression may have oncogenic or tumor-suppressive effects.

In detail, You et al. [44] found that low levels of expression of miR-495 were correlated to an increase in EMT, proliferation and migration of CSCs. Similarly, according to Yu et al. [45], the low interference of miR-204 with its targets, *SOX4* and *SLUG*, resulted in an increase in stemness, EMT process and proliferation of CSCs.

On the other hand, abnormally high expression of miR-146 was seen for the activation of CD24-AKT-β-catenin signaling, which significantly enhanced CSCs proliferation in vitro and tumorigenic ability in vivo [46].

Oncogenic properties in CSCs OSSC were reported for miR-21-3p, miR-18a-3p, miR-210-3p, miR-155-5p, miR-181a-5p and miR-19a-3p; their elevated levels were associated with ADAR1 overexpression, which was, in turn, physically interacting with DICER, a key cytoplasmic component for miRNAs elaboration [47].

Two of the above-mentioned miRNAs (miR-185 and miR-101-3p) have been proposed as biomarkers and/or potential options for therapeutic application.

Cetin et al. [48] referred to a study dealing with the effect of engineered MSC-EVs with a high copy number of miR-185 on OSCC development. In an induced model, treatment with miR-185-MSC-EVs reduced the proliferative and angiogenesis markers, and miR-185-EV treatment activated the apoptotic pathway through direct targeting of *AKT*, an upstream regulator of caspase-9. Intratumoral administration of miR-101-3p modified human primary BM-MSC EVs in *BALB/c* nude mice with SCC resulted in a decrease in *COL10A1* expression, with a consequent reduction of tumor volume and weight and the invasion, migration and colony-forming ability of the cells [48]. These two studies show that treatments with miRNA-loaded MSC-derived EVs could counteract HNSCC.

Regarding SCC, conclusions concerning miRNAs related to EVs or to CSCs are almost dissimilar. All the manuscripts referring to MSCs-EVs miRNAs describe a scenario characterized by inhibition of proliferation, invasion, migration and, at last, tumor progression. It is important to note that the tumor suppressive role described for miR-101-3p has been recognized both for HNSCC and OSCC and by two authors, while other miRNAs have been cited just once. It has also gained attention as a therapeutic tool. Finding conclusive correlations between miRNA and CSCs is more complex; according to the different types of SSC (oral, head and neck, cutaneous), diverse miRNAs have been considered, and supporting as well as suppressive tumor features have been described. In general, for approximately half of the 28 considered miRNAs, the increased proliferation, migration and EMT observed in CSCs was the result of low interference with miRNAs, while for the other portion, it was the abnormally high expression of miRNAs that support tumor progression.

### 2.2. Inflammatory Skin Diseases

#### 2.2.1. Psoriasis

The role of miRNAs in the pathogenesis of psoriasis is still unclear, although previous studies have indicated miR-146a-5p as a blood-based biomarker able to foresee the therapeutic response to systemic treatment [49,50].

Additionally, MSCs have been demonstrated to be involved in pathological processes associated with psoriasis [51,52], and, in turn, their immunophenotype may be influenced by systemic treatments [53,54].

Previous studies reported that psoriatic dermal MSCs stimulated keratinocyte (KC) proliferation [55].

Liu et al., in 2019 [56], evaluated the miRNA expression profile of MSCs from psoriatic skin lesions and investigated possible mechanisms associated with psoriasis. In total, out of 6323 miRNAs detected, only miR-17-5p, miR-30e-5p and miR-142-3p/5p have been found differentially expressed in psoriatic lesions, with miR-142-3p specifically involved in epidermal inflammation.

Wang et al., in 2018 [57], aimed to clarify the pathogenetic role of miR-31 in dermal MSCs in psoriasis.

The authors found a reduced miR-31 expression in MSCs derived from patients with psoriasis compared to healthy controls, and they speculated that the low expression of miR-31 may induce psoriasis by the modulation of immunity through the aberrant expression of some of miR-31 target genes in the MSCs of patients with psoriasis [57].

Nowadays, psoriasis is considered more than skin deep, and its systemic nature is widely recognized, together with the evidence of metabolic abnormalities’ coexistence. Several studies have already suggested the linking role of chronic inflammation between psoriasis and metabolic abnormalities. Several miRNAs play a crucial regulatory role in promoting inflammation.

Firstly, Hou et al. found that the pro-inflammatory miRNA miR-155 was significantly overexpressed in psoriatic MSCs (2.44-fold). Therefore, the authors suggested that the elevation in miR-155 levels may be an indicator of, or a key regulatory pathway in, the pathogenesis of psoriasis, resulting in functionally impaired dermal MSCs by a detrimental ability to suppress the inflammatory response [58].

Recently, Liu et al. [59] confirmed the potential role of miR-155 in psoriasis pathogenesis through its upregulation in psoriasis, promoting proliferation, migration, inflammatory response and metabolite levels of MSCs, while inhibiting apoptosis [59]. In comparison with MSCs obtained from healthy individuals, the authors demonstrated that the glycolysis levels were increased in MSCs obtained from psoriasis patients and this increase was related to miR-155 expression through the negative regulation of the TP53INP1/p53 signaling pathway.

Only one manuscript specifically dealt with the effect of MSCs-derived miRNAs on keratinocytes. Li et al. evaluated the expression of several different miRNAs in normal human epidermal keratinocyte cells (NHEK) cocultured with either psoriatic or control MSCs. Expression levels of miRNA were determined by RNA sequencing [60].

A coculture of NHEK with psoriatic MSCs induces 32 differentially expressed miRNAs, in which the expression levels of miR-1 increased approximately 16-fold over control MSCs-incubated NHEK. Likewise, expression levels of miR-21-3p increase more than twofold.

These miRNAs are directly associated with the increase of *IL6*, *VEGF* and enhanced proliferation of HaCaT cells. In addition, phototherapy improves psoriasis, accompanied by a reduction in miR-21 expression in psoriatic lesions. Furthermore, miR-21 and miR-1 regulate intercellular calcium homeostasis, which is linked to the senescent phenotype of KC in psoriasis.

Hawkes et al. [61] reviewed the aberrant miRNAs in psoriasis produced by keratinocytes.

Among them, only miR-203 showed a correlation with MSCs. On one hand, its overexpression was correlated with a decrease in the suppression of cytokine signaling 3 (*SOCS3*) and subsequent elevation in signal transducer and activator of transcription-3 (*STAT3*), a transcription factor in keratinocytes that is fundamental to the development of psoriatic skin lesions; on the other hand, miR-203 drove the self-renewing, stratification and differentiation capacity of stem cells located within the innermost basal layer of the epidermis.

In the context of psoriasis, some specific miRNAs derived from dermal MSCs have already been identified and all of them sustain the maintenance of an inflamed microenvironment followed by metabolic abnormalities, excessive immune cell infiltration and keratinocyte proliferation.

#### 2.2.2. Atopic Dermatitis

The role of miRNA expression in AD is far from being revealed. To the best of our knowledge, only one study dealt with this issue.

The authors investigated the effect of human adipose MSCs on AD in a *BALB/c* mouse model. The mouse miR-122a-5p mimic inhibited AD, while the suppression of cytokine signaling 1 (*SOCS1*), a predicted downstream target of miR-122a-5p, was required for AD [62]. Further studies focusing on miRNA profiles in MSCs are required.

## 3. Materials and Methods

According to the approach developed by Arksey and O’Malley [28], five essential steps were performed: identification of the research question; identification of appropriate studies; selection of studies; tracking of data; and collection, summarization and reporting of results. The Preferred Reporting Items for Systematic Reviews and Meta-Analysis (PRISMA) extension for scoping review criteria was used to guide the conduction and reporting of the review [63]. This review was registered in the International Prospective Register of Systematic Reviews (PROSPERO) with the registration number “CRD42023420245”.

### 3.1. Identification of the Research Question

The research question was identified by a brainstorming approach that involved the entire research team, consisting of 3 dermatologists with expertise in the research field of inflammatory and neoplastic skin disorders, 2 histologists and one pharmacologist with experience in the research field of MSCs and miRNAs.

During this initial meeting, the group identified the research question and, subsequently, determined the research strategy. The research question was: “whether and how can miRNAs dysregulation affect the behavior of MSCs, driving the onset of skin disorders?”

### 3.2. Study Selection Process

A worldwide systematic review of studies reporting on this topic was performed, using 3 electronic medical databases—PubMed, Web of Science and SCOPUS—considering articles dated after 1 January 2012.

The search terms were selected to identify studies describing the relation among miRNAs, MSCs and inflammatory/neoplastic skin disorders.

The keywords used were:

1. “miRNAs AND human mesenchymal stromal cells AND melanoma”
2. “miRNAs AND human mesenchymal stromal cells AND squamous cell carcinoma”
3. “miRNAs AND human mesenchymal stromal cells AND basal cell carcinoma”
4. “miRNAs AND human mesenchymal stromal cells AND psoriasis”
5. “miRNAs AND human mesenchymal stromal cells AND atopic dermatitis”

All selected databases were searched from their respective inceptions on 12 October 2022. In addition, we searched, by hand, the reference lists of other relevant articles on MSCs and psoriasis.

In this first phase, for neoplastic skin disorders, 101 records for “miRNAs AND human mesenchymal stem cells AND squamous cell carcinoma”; 56 records for “miRNAs AND human mesenchymal stromal cells AND melanoma”; and 5 records for “miRNAs AND human mesenchymal stromal cells AND basal cell carcinoma” were retrieved from the selected databases.

For inflammatory skin disorders, results were less encouraging, with 9 results for “miRNAs AND human mesenchymal stromal cells AND psoriasis” and 1 for “miRNAs AND human mesenchymal stromal cells AND atopic dermatitis”. A total of 172 were retrieved globally (Table 1).

Records after duplicate removal totaled 46 for “miRNAs AND human mesenchymal stromal cells AND melanoma”, 80 for “miRNAs AND human mesenchymal stromal cells AND squamous cell carcinoma” and 3 for “miRNAs AND human mesenchymal stromal cells AND basal cell carcinoma”. Among inflammatory diseases, removing duplicates led to having 6 records for “miRNAs AND human mesenchymal stromal cells AND psoriasis”, and 1 for “miRNAs AND human mesenchymal stromal cells AND atopic dermatitis”.

Among the selected records, none were marked as ineligible by automation tools. Relevant studies were then chosen. This process occurred in three phases. In the first phase, 2 researchers (MDV, AC) independently selected articles based on title, reducing the items to 14, 36, and 0, respectively, consistent with “miRNAs AND human mesenchymal stromal cells AND melanoma”, “miRNAs AND human mesenchymal stromal cells AND squamous cell carcinoma” and “miRNAs AND human mesenchymal stromal cells AND basal cell carcinoma” for neoplastic diseases.

For inflammatory and immune-mediated skin diseases, selection based on title reduced records to 7 and 1 for “miRNAs AND human mesenchymal stromal cells AND psoriasis” and “miRNAs AND human mesenchymal stromal cells AND atopic dermatitis”, respectively, for a total of 57 records included. Any disagreements were resolved by consulting a senior researcher (MO).

In the second phase, abstracts were evaluated. All members of the research team independently evaluated each abstract. The research group resolved all discrepancies through unanimous consent. Five articles were excluded and 9 were evaluated for full-text analysis for “miRNAs AND human mesenchymal stromal cells AND melanoma” and 21 were excluded and 15 evaluated for “miRNAs AND human mesenchymal stromal cells AND squamous cell carcinoma”. Among inflammatory diseases, none of selected reports were excluded from the analysis.

The third phase consisted of a critical appraisal of the full texts of the 9 and 15 selected papers for melanoma and non-melanoma skin diseases, and the 8 papers related to inflammatory diseases. To be included in our review, studies had to be focused on the research question. All included studies had to be published with abstracts available. No restrictions on study design were considered, and in vitro and in vivo pre-clinical trials, controlled clinical trials, case-control studies, cross-sectional studies and case series were included. Nevertheless, neither cross-sectional studies nor case-control studies were found among the selected papers.

Finally, 9 and 13 papers were, respectively, included for melanoma and non-melanoma skin diseases, and 7 records were included for inflammatory and immune-mediated skin diseases.

This selection process immediately highlighted two limitations of this topic: the use of “mesenchymal” among keywords led to many unfitting results, indicating an abuse of this word in different contexts; for other manuscripts, the reported skin disease has been taken as an example only without further consideration.

Often, the word “mesenchymal” refers to Epithelial–Mesenchymal Transition (EMT); even if the EMT is a mechanism used by cancer cells to move and metastasize, it was out of context in this scoping review focused on mesenchymal stromal cells. For this reason, 2 manuscripts on melanoma [64,65], 9 on SCC [66,67,68,69,70,71,72,73,74] and 1 on BCC [75] were excluded.

Nine other manuscripts were rejected because they focused on different pathologies and referred to melanoma/SCC/BCC only to mention one example [76,77,78,79,80,81,82,83,84].

### 3.3. Data Extraction

A data extraction module was designed by A.C. before data extraction to accelerate the entire process. To answer the research question, the following information was extracted from the included articles: Author(s) name(s) and publication date; study design; study population; sample size; measured outcomes; study results; and study recommendations.

## 4. Conclusions

This scoping review is focused on the existing literature on how miRNA dysregulation might affect the properties of MSCs and, subsequently, drive the onset of skin disorders (both neoplastic and inflammatory). The 27 full-length papers selected draw a complex puzzle of cellular mechanisms related to miRNA-MSCs-skin disorders (Table 2) and underline the ambivalent biologic behavior of MSCs, characterized by anti-inflammatory/pro-inflammatory–tumor supporting/suppressive properties.

A limitation of this study is related to the lack of knowledge on the subject: even if the search in the database initially produced 171 items, most of them were completely unsuitable for keywords with improper use of the word “mesenchymal”, which compromised the research. Furthermore, the novelty of the theme is also evident in the lack of a solid hypothesis: the diverse manuscripts often use completely different approaches, giving some insights like beacons in the night. This topic is taking its first steps and in the future, all the mechanisms that actually are only speculations will certainly find new evidence and, for some miRNAs, the specific assignment to roles as biomarkers or as therapeutic tools is very close.

## Figures and Tables

**Table 1:** Number of records found in the electronic medical databases—PubMed, Web of Science, SCOPUS according to the keywords.

	N. Records		
	PubMed	Web of Science	Scopus
Melanoma	15	18	23
Squamous cell carcinoma	36	26	39
Basal cell carcinoma	1	1	3
Psoriasis	6	1	2
Atopic dermatitis	1	0	0

**Table 2:** List of the selected manuscripts, identified miRNAs and their main functions.

	Author(s) Name	Publication Date, Type	doi	miRNA	Function
MELANOMA	Wang X. et al. [29]	2022 OA	10.3233/CBM-210409	miR-138-5p	miR-138-5p targets the stem gene *SOX4* and its high expression in MSC-derived EVs increases cell apoptosis
	Yang Y. et al. [30]	2022 OA	10.1038/s41417-021-00392-8	miR-374a-5p	miR-374 expression is inhibited by EVs derived from BM-MSCs carrying *NEAT1*; its inhibition is connected to the M2 polarization of macrophages and the occurrence/development of melanoma
	Gyukity-Sebestyén E et al. [31]	2019 OA	10.3389/fimmu.2019.02459	More than 120 miRNAs inside EVs	miRNAs and proteins inside the melanoma EVs can re-educate multipotent tissue-derived MSCs, forming a melanoma-like MSC^PD−1+^ subpopulation promoting tumor progression
	Sahranavardfard P. et al. [32]	2021 OA	10.22074/cellj.2021.7311	miR-205, -203, -9, and -15b	miR-205, -203, -9 and -15b promote stemness pluripotency, proliferation and EMT of malignant cells
	Fomeshi RL et al. [33]	2015 OA	10.1515/cmble-2015-0025	miR-10b, 21, 200c, 373 and 520c	miR-10b, 21, 200c, 373 and 520c are upregulated in melanospheres
	Sahranavardfard P et al. [34]	2019 OA	10.1002/jcp.28619	miR-203	miR-203 reinforces the ability of proliferation, colony and spheres formation, migration and tumorigenesis in melanoma cell lines
	Whang Y. et al. [35]	2021 OA	10.1080/15476286.2021.1950463	miR-381-3p	Sequestering miR-381-3p leads to an increase in cell proliferation, migration and stemness in melanoma via the *ZBED3-AS1/ARID4B* axis
	Divisato G. et al. [36]	2021 R	10.3390/biom11081074	miR-29a	miR-29a mediates antiproliferative effects by downregulating *CDK6*, a regulator of the G1/S phase
	Maadi H. et al. [37]	2016 OA	10.1016/j.biocel.2016.11.004	miR-302	miR-302 inhibits proliferation, angiogenesis and invasion through the MET process, leading to a reduction in tumorigenic potential
SQUAMOUS CELL CARCINOMA	Wang X. et al. [38]	2021 R	10.1186/s13046-021-01840-x.	miRNA-34c, 101-3p, 185	MSCs-derived EVs overexpressing miRNA-34c inhibit invasion, migration, proliferation in HNSCC, as do miRNA-101-3p and 185 in OSCC
	Panvongsa W. et al. [39]	2022 R	10.3390/cancers14051160	miRNA-101-3p, 185	BM-MSCs-derived EVs overexpressing miR-101-3p or miR-185 suppress cancer cell proliferation and tumor growth both in vitro and in vivo in HNSCC
	Fitriana M et al. [40]	2021 R	10.3390/cancers13071742	miRNA-125, 134, 196b, 19a, 424, 106A-5p, 629-3p miRNA let-7, 520b	miRNA-125, 134, 196b, 19a, 424, 106A-5p and 629-3p act as oncomirs in HNSCC. miRNA let-7 family activates the expression of *POU5F1*, *NANOG* and *SOX2*, suppresses tumor formation and metastasis. miRNA-520b inhibits EMT and sensitizes cells to chemoradiotherapy through suppression of *CD44*
	Tu H. et al. [41]	2013 R	10.1097/MOO.0b013e32835e1d6e	miR-21	miR-21 is oncomir and regulates stemness and the epithelial–mesenchymal transition of tumour cells
	Subramanian C. et al. [42]	2017 OA	10.1016/j.neo.2017.09.003	miRNA-15b, 16, 17, 107,20a, 106b, 128, 425, 223, 299, 145, 302a, 494, 409, and 128	miRNA-15b, 16, 17, 107,20a, 106b, 128, 425, 223, 299, 145, 302a, 494, 409 and 128 are implicated in CSCs, improving self-renewal, differentiation, metastasis development and tumor recurrence
	Bai XP et al. [43]	2018 OA	10.1002/jcb.26379	miR-142-5p	The expression of miR-142-5p can promote the expression of stem markers, such as *CD44*, *SOX2*, *NANOG* and *OCT4*
	You X. et al. [44]	2020 OA	10.1186/s13287-020-1576-3	miR-495	miR-495 targets CSCs and its expression is inversely correlated to EMT, proliferation and metastasis in OSSC
	Yu CC. et al. [45]	2016 OA	10.18632/oncotarget.7745	miR-204	miR-204 binds on the 3′UTR-regions of *SOX4*, suppressing the stemness of the tumor and therefore its growth in OSCC
	Ghuwalewala S et al. [46]	2021 OA	10.3389/fonc.2021.651692	miR-146a	miR-146a enhances stemness phenotype and self-renewal capacity in OSCC and HNSCC
	Liu X. et al. [47]	2019 OA	10.1186/s13046-019-1300-2	miR-21–3p, miR-18a-3p, miR-210-3p, miR-155-5p, miR-181a-5p e miR-19a-3p	miR-21–3p, miR-18a-3p, miR-210-3p, miR-155-5p, miR-181a-5p e miR-19a-3p regulate the stemness of OSCC cell lines affecting the expression of *SOX2* and *POU5F1* via *ADAR1*
	Cetin Z. et al. [48]	2021 R	10.1007/s12015-020-10082-x	miR-185, 101-3p	Inhibition of proliferation and angiogenesis as well as of *COL10A1* expression have been reported for MSC-EVs loaded, respectively, with miR-185 or miR-101-3p
PSORIASIS	Liu R. et al. [56]	2019 OA	10.1684/ejd.2018.3483	miR-17-5p, 30e-5p, miR-142-3p/5p	miR-17-5p, 30e-5p, miR-142-3p/5p are differentially expressed in psoriatic vs healthy skin MSCs and are related to inflammation
	Wang Q. et al. [57]	2019 OA	10.1111/ijd.14197	miR-31	The reduced expression of miR-31 in dermal MSCs participates in psoriasis onset facilitating the activation of T lymphocytes
	Hou R.X. et al. [58]	2016 OA	10.4238/gmr.15038631	miR-155	miR-155 is overexpressed in psoriatic MSCs and it is related to increased inflammation
	Liu Z. et al. [59]	2022 OA	10.1016/j.jdermsci.2022.02.001	miR-155	miR-155 promotes glycolysis in psoriatic MSCs causing their metabolic abnormalities
	Li X. et al. [60]	2020 OA	10.1111/1346-8138.15369	miR-21-3p and miR-1	Keratinocytes, after co-cultures with psoriatic MSCs, overexpress miR-21-3p and miR-1, which are associated to increased levels of *IL6*, *VEGF* and enhanced proliferation
	Hawkes JE. et al. [61]	2016 R	10.1038/JID.2015.409	miR-203	miR-203 regulates self-renewing and differentiation capacity of stem cells located within the basal layer of the epidermis
ATOPIC DERMATITIS	Kim M. et al. [62]	2018 OA	10.3389/fphar.2018.01175	miR-122a-5p	miR-122a-5p acts as a negative regulator of *SOCS1* that, together with *CXCL13*, causes the activation of macrophages and T cells responses during AD

OA: Original Articles; R: Review; MSCs: Mesenchymal Stromal Cells; EVs: Extracellular Vesicles; EMT: Epithelial to Mesenchymal Transition; MET: Mesenchymal to Epithelial Transition; BM-MSCs: Bone Marrow derived MSCs; HNSCC: Head and Neck Squamous Cell Carcinoma; OSCC: Oral Squamous Cell Carcinoma; CSCs: Cancer Stem Cells; AD: Atopic Dermatitis.

## Data Availability

No new data were created or analyzed in this study. Data sharing is not applicable to this article.

## Supplementary Materials

The following supporting information can be downloaded at: https://www.mdpi.com/article/10.3390/ijms24108502/s1.
