# Supplementary material for: miRNAs, Mesenchymal Stromal Cells and Major Neoplastic and Inflammatory Skin Diseases: A Page Being Written: A Systematic Review
Source: Int J Mol Sci. 2023 May 9;24(10):8502. doi: 10.3390/ijms24108502 (PMC10217999; doi:10.3390/ijms24108502)
Supplement: Supplementary file 1 [file ijms-24-08502-s001.zip › ijms-2273706-supplementary.pdf]

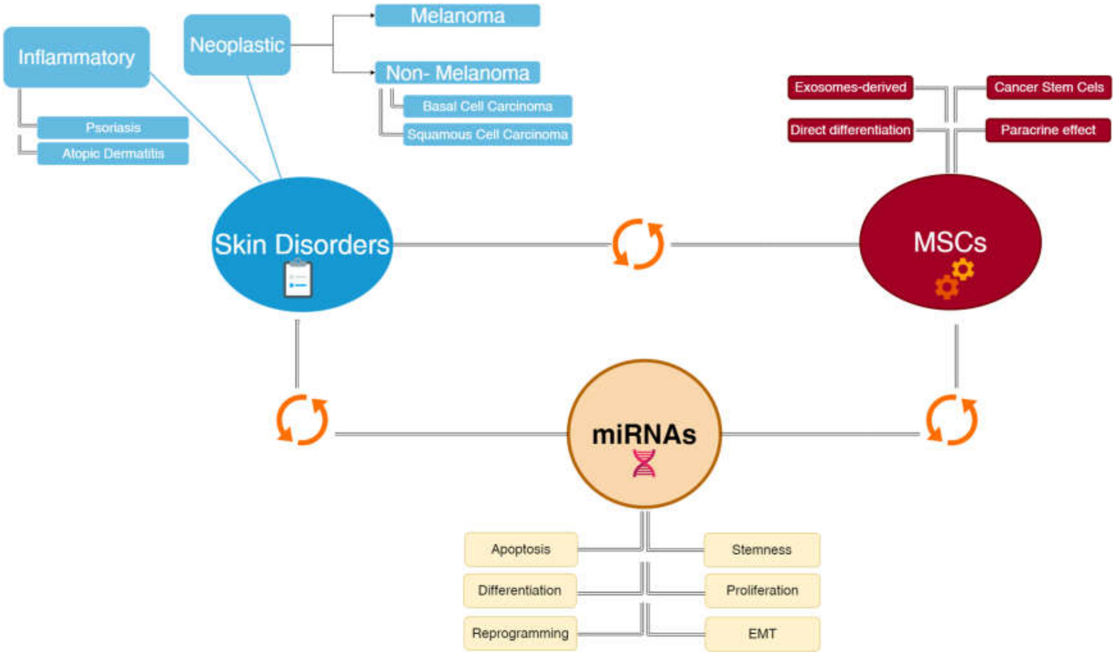

Figure S1. The image describes the complex, multidirectional relationship among skin disorders, miRNAs and Mesenchymal Stromal Cells (MSCs). In the boxes, the classification of skin disorders and the main cellular pathways driven by MSCs and miRNAs. https://app.diagrams.net/.
